# Impact of Parenteral Glutamine Supplement on Oncologic Outcomes in Patients with Nasopharyngeal Cancer Treated with Concurrent Chemoradiotherapy

**DOI:** 10.3390/nu14050997

**Published:** 2022-02-26

**Authors:** Chih-Chun Wang, Tzer-Zen Hwang, Chuan-Chien Yang, Ching-Feng Lien, Chien-Chung Wang, Yu-Chen Shih, Shyh-An Yeh, Meng-Che Hsieh

**Affiliations:** 1 Department of Otolaryngology, E-Da Hospital, Kaohsiung 82445, Taiwan; philips115@gmail.com (C.-C.W.); med9001115@yahoo.com.tw (T.-Z.H.); srbmrg.tw@yahoo.com.tw (C.-C.Y.); cphtem44@gmail.com (C.-F.L.); philips115@isu.edu.tw (C.-C.W.); 2 College of Medicine, I-Shou University, Kaohsiung 82445, Taiwan; 107033@dhp.kh.edu.tw (Y.-C.S.); 109046@dhp.kh.edu.tw (S.-A.Y.); 3 Department of Otolaryngology, E-Da Cancer Hospital, Kaohsiung 82445, Taiwan; 4 Department of Radiation Oncology, E-Da Hospital, Kaohsiung 82445, Taiwan; 5 Department of Hematology-Oncology, E-Da Cancer Hospital, Kaohsiung 82445, Taiwan

**Keywords:** nasopharyngeal carcinoma, chemoradiotherapy, glutamine, dipeptiven, oncologic outcomes, oral mucositis

## Abstract

Background: Oral mucositis (OM) is a common toxic side effect in nasopharyngeal carcinoma (NPC) patients receiving concurrent chemoradiotherapy (CCRT) that has a negative impact on treatment outcomes and patients’ survival. Our study aimed to evaluate the impact of parenteral glutamine supplement (dipeptiven) on oncologic outcomes in patients with NPC treated with CCRT. Methods: Patients who were diagnosed with pathologically proved NPC and treated with CCRT were enrolled into our study. Patients were classified as dipeptiven (+) and dipeptiven (–). Oncologic outcomes were measured, and multivariate regression analysis was performed. Grade 3–4 treatment related toxicities were also documented. Results: A total of 144 patients with NPC were recruited in this study to evaluate oncologic outcomes, with 41 dipeptiven (+) and 103 dipeptiven (–). CCRT interruption rate and severe adverse effect (SAE) rate were significant lower in the dipeptiven (+) group than in the dipeptiven (–) group. The median overall survival (OS) was not mature yet in the dipeptiven (+) group and 30 months in the dipeptiven (–) group (*p* < 0.01). Multivariate analysis demonstrated that dipeptiven supplementation and CCRT interruption were independent predictors associated with better survival. The OS was longest in patients with a dipeptiven supplement and patients who had CCRT interruption had significantly worst OS. As for safety profiles, grade 3 to 4 adverse effects were fewer in dipeptiven (+) than in dipeptiven (–). Conclusion: Dipeptiven supplementation is crucial in NPC patients treated with CCRT, which can ameliorate treatment-related toxicity and augment treatment efficacy. Further prospective clinical trials are warranted to validate our results.

## 1. Introduction

Nasopharyngeal carcinoma (NPC) is a malignancy arising from the nasopharyngeal mucosa, which is most common in east and southeast Asia. According to the International Agency for Research on Cancer in 2018, more than 129,000 new cases of NPC were diagnosed, accounting for only 0.7% of all cancers in 2018 [1,2]. Most new cases are in China, with an age-standardized rate of 3 per 100,000. According to current guidelines, chemotherapy combined with radiotherapy is a crucial treatment for locoregionally advanced NPC [3]. Previous trials have demonstrated that concurrent chemoradiotherapy (CCRT) has better prognosis than radiotherapy alone in locoregionally advanced NPC [4,5]. However, severe adverse effects of CCRT might compromise the survival benefits. Oral mucositis (OM) is one of adverse events in NPC patients receiving CCRT that has a negative impact on treatment outcomes and patients’ survival [6]. Previous literature reported that the incidence of oral mucositis was very high during CCRT for head and neck cancer, accounting for 67.7% [7]. Approximately 40% of patients with NPC who receive chemotherapy and 100% of patients who receive CCRT develop OM [8]. OM has a negative impact in our patients, including severe oral pain, debilitating clinical situations, malnutrition due to eating difficulty, and increased rates of oral infection [9]. Significant nutritional deficiency leads to body weight loss, impaired wound healing, and decreased resistance to infection, as well as deteriorated quality of life [10]. Treatment of OM is focused on symptom relief, pain alleviation, complication prevention, and oral hygiene maintenance [11]. Several strategies have been investigated for treatment of OM, including anti-inflammatory drugs, antibiotics, anti-fungal drugs, corticosteroids, painkillers, amino acids, vitamins, and other agents [12,13,14]. However, treatment guidelines regarding prophylaxis and management of OM in NPC patients receiving chemoradiotherapy are not well-established.

Dipeptiven (Fresenius Kabi AG, Taiwan, 100 mL, 20%) is a parenteral amino acid solution containing dipeptide alanyl-glutamine. L-Glutamine is the most abundant and conditional amino acid in human blood [15]. It is also an amino acid precursor for protein synthesis and cell proliferation, and it is a precursor for nucleotides, glutamate, and glutathione synthesis [16]. The serum concentration of glutamine is usually exhausted in the face of stress, such as radiotherapy. In that case, a glutamine supplement might be beneficial for preventing mucositis in patients at high risk, especially CCRT. Glutamine supplements may repair cellular injury and force recovery, which might decrease the probability and severity of OM. However, all of the previous literature has focused on the effect of glutamine supplements for attenuating treatment-related adverse effects, as well as OM. To date, little was known about whether glutamine supplements also influence oncologic outcomes in cancer patients. Thus, we conducted a retrospective study to evaluate the impact of dipeptiven supplementation on oncologic outcomes in patients with NPC treated with CCRT.

## 2. Materials and Methods 

### 2.1. Patients 

Patients who were at an age older than 18 years and diagnosed with pathologically proved NPC from 2018 to 2021 at E-Da Hospital and E-Da Cancer Hospital were retrospectively reviewed. Patients who were treated with CCRT were enrolled into our study. Patients were classified as dipeptiven (+) if they received a dipeptiven supplement during CCRT, while patients were classified as dipeptiven (–) if they never received a dipeptiven supplement during CCRT. All the patients’ basic characteristics were collected by chart review. Exclusion criteria included incomplete CCRT, irregular evaluation intervals, and being lost to follow-up. Our study was approved by the E-Da Hospital Institutional Review Board (EMPR-109-012) and was conducted in accordance with the Declaration of Helsinki. Given that this was a retrospective observational study, informed consent was exempted. 

### 2.2. Treatments 

All patients in our study received CCRT with weekly cisplatin and conventional radiotherapy. The principles of chemotherapy and radiotherapy followed our treatment guideline. For details, chemotherapy was administrated with a 1-week cycle of cisplatin 30–35 mg/m^2^, and fractioned radiotherapy was given with 70 Gy in 35 fractions over 7 weeks. In the parenteral glutamine supplement group, dipeptiven was administrated at least one bottle per week during the period of CCRT. If patients developed severe OM, dipeptiven could be administrated twice or thrice per week at the physician’s discretion until amelioration of OM or complete radiotherapy course. Induction chemotherapy and adjuvant chemotherapy were all allowed in this study. Image studies were arranged periodically to evaluate the treatment response after CCRT. 

### 2.3. Statistical Analysis

All basic characteristics were retrospectively retrieved from a medical chart review and presented with frequencies. Chi-square tests were calculated to analyze the differences between dipeptiven (+) and dipeptiven (–). Oncologic outcomes were presented with CCRT interruption rate, severe adverse effects (SAE) rate, overall response rate (ORR), disease control rate (DCR), and overall survival (OS). The definition of CCRT interruption indicated discontinuation of chemotherapy or radiotherapy due to intolerance of adverse effects. SAE referred to any treatment-related adverse effects leading to hospitalization. Objective response was determined according to the RECIST 1.1 guidelines, including complete response (CR), partial response (PR), stable disease (SD), and progressive disease (PD). Overall survival (OS) was measured from the diagnosis of nasopharyngeal carcinoma until the date of death or last visit. Kaplan–Meier analysis was also performed for survival. Multivariate Cox regression analysis with “enter” selection was also conducted to adjust for potential confounders. All *p* values were two-sided and were considered significant if *p* values < 0.05. Grade 3–4 treatment-related adverse events were recorded according to the National Cancer Institute’s Common Terminology Criteria V3.0.

## 3. Results

### 3.1. Patients Characteristics

In total, 144 NPC patients were recruited into our study to evaluate oncologic outcomes. Median follow-up period was 20 months, and the median age of our patients was 53 years. Table 1 summarizes the basic characteristics of our patients. In general, most patients were male in gender (73.6%) and younger than 60 years (70%). Up to 85% of patients had fit Eastern Cooperative Oncology Group Performance Status (ECOG PS) 0–1, 62% of patients had body mass index (BMI) > 24 kg/m^2^, 85% of patients had weight loss ≦ 5% after complete CCRT, and 95% of patients had adequate renal function with creatinine clearance rate (CCr) higher than 60 mg/mL. Most patients had a locally advanced stage, with 82% stage III–IVA. In addition to CCRT, 30% of patients received induction chemotherapy and 75% of patients received adjuvant chemotherapy after CCRT. After stratification by dipeptiven supplement, 41 patients were dipeptiven (+) and 103 patients were dipeptiven (–). Baseline characteristics of patients in these two arms were balanced, including gender, age, ECOG PS, BMI, weight loss, CCr, clinical T stage, clinical N stage, initial stage, induction chemotherapy, and adjuvant chemotherapy.

### 3.2. Survival Outcomes

**Figure 1:**
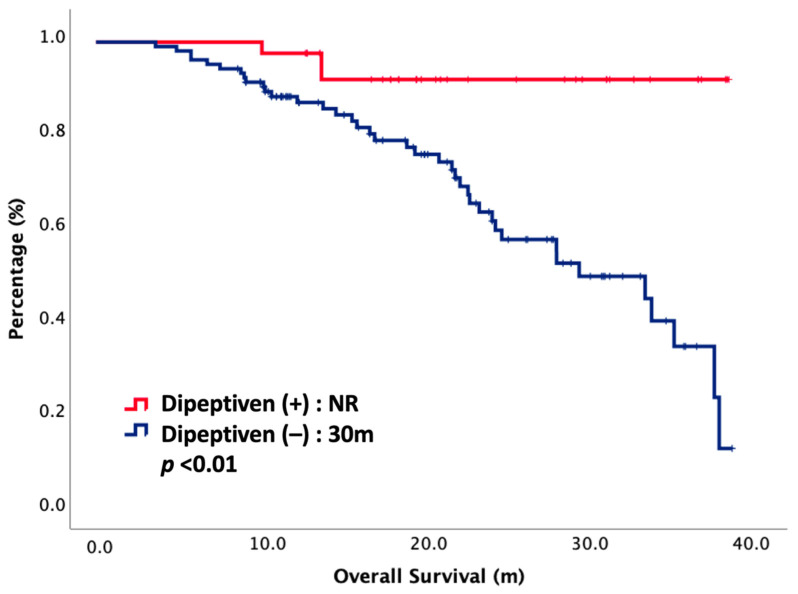
Overall survival of 144 NPC patients treated with CCRT, stratified by dipeptiven. NR, not reached.

At the cut-off date of our study, only 29% patients had died. The oncologic outcomes between dipeptiven (+) and dipeptiven (–) are presented in Table 2. The toxicity of CCRT was improved under dipeptiven support. The CCRT interruption rate was significantly lower in the dipeptiven (+) group than in the dipeptiven (–) group, accounting for 0% and 21%, respectively (*p* < 0.01). The SAE rate was also significantly lower in the dipeptiven (+) group than in the dipeptiven (–) group, accounting for 0% and 12%, respectively (*p* = 0.02). However, ORR and DCR did not have significance in either arm. The ORR and DCR values were 100% vs. 90% and 100% vs. 96% in the dipeptiven (+) vs. dipeptiven (–) group, respectively. Notably, the CR rate was significant in dipeptiven (+) as compared with dipeptiven (–), accounting for 78% vs. 57%, respectively (*p* = 0.02). The median OS was not reached in the dipeptiven (+) group, and it was 30 months in the dipeptiven (–) group (*p* < 0.01). The survival curve is plotted in Figure 1.

### 3.3. Multivariate Regression Analysis

**Figure 2:**
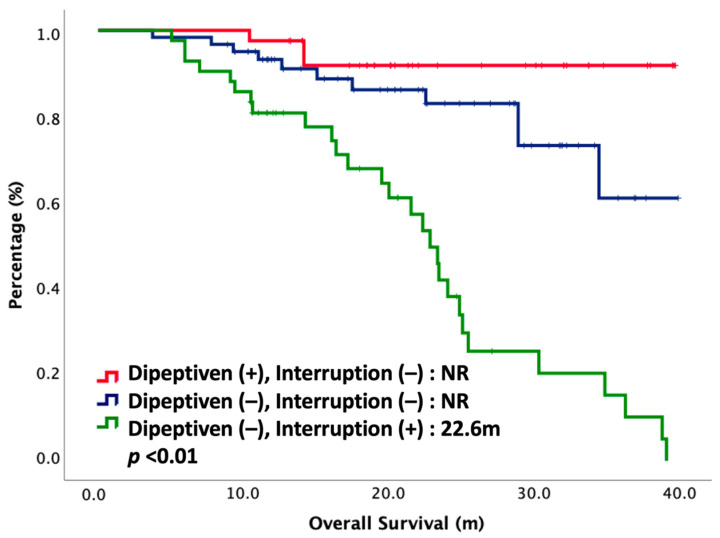
Overall survival of 144 NPC patients treated with CCRT, stratified by dipeptiven and CCRT interruption. NR, not reached.

Cox regression analyses with survival for potential prognostic factors were performed. Hazard ratios (HR) with 95% CI (confidence interval) are depicted in Table 3. Univariate analysis showed BMI (HR: 0.48, 95% CI: 0.26–0.89, *p* = 0.02), induction chemotherapy (HR: 0.10, 95% CI: 0.01–0.74, *p* = 0.02), adjuvant chemotherapy (HR: 0.21, 95% CI: 0.11–0.40, *p* < 0.01), dipeptiven supplement (HR: 0.13, 95% CI: 0.04–0.44, *p* < 0.01), and CCRT interruption (HR: 0.15, 95% CI: 0.08–0.29, *p* < 0.01) were strongly correlated with OS. Multivariate analysis demonstrated that dipeptiven supplementation (HR: 0.31, 95% CI: 0.09–0.95, *p* = 0.04) and CCRT interruption (HR: 0.32, 95% CI: 0.15–0.68, *p* < 0.01) were independent predictors associated with better survival. Patients were then stratified according to these two predictive markers: dipeptiven supplement and CCRT interruption. OS was significantly different between each group. The OS curve of each group is plotted in Figure 2. The OS was longest in patients with dipeptiven supplementation, followed by patients without dipeptiven supplementation. Patients who had CCRT interruption had significantly worse OS.

### 3.4. Safety Profiles

As for safety profiles, OM remained the major concern among NPC patients receiving CCRT. In general, 90% patients in the dipeptiven (+) group and 92% patients in the dipeptiven (–) group developed all-grade OM. For grade 1 and 2 OM, there were 35 (85%) patients in the dipeptiven (+) group and 74 (72%) patients in the dipeptiven (–) group. For grade 3 and 4 OM, there were 2 (5%) patients in the dipeptiven (+) group and 21 (20%) patients in the dipeptiven (–) group. Table 4 discloses all grade 3 to 4 treatment-related adverse events (AE). Grade 3–4 AEs were significantly different between the two treatment arms. In general, the incidence of grade 3–4 AEs was lower in dipeptiven (+) than in dipeptiven (–). As for hematologic events, 2% of patients in dipeptiven (+) and 16% of patients in dipeptiven (–) had grade 3–4 neutropenia (*p* = 0.02). There were insignificant in grade 3–4 febrile neutropenia and anemia in both arms. As for non-hematologic events, grade 3–4 fatigue (*p* = 0.02), oral mucositis (*p* = 0.02), and peripheral neuropathy (*p* = 0.03) were significantly fewer in dipeptiven (+) than in dipeptiven (–). 

## 4. Discussion

This observational study demonstrated that dipeptiven supplementation was essential for NPC patients treated with CCRT. The dipeptiven supplement diminished the treatment-related adverse effects, decreased CCRT interruption rate, increased CR rate, and also improved the prognosis. There are several explanations regarding why dipeptiven supplementation improved survival. The major reason was the dipeptiven supplement could help to reduce the severity of leukopenia and oral mucositis, resulting in better tolerance of CCRT in patients with NPC. Once patients became more tolerable, the CCRT interruption rate was decreased. With a lower CCRT interruption rate, the CR rate was increased and the outcomes became better. This result was consistent with a previous publication. Xu et al. retrospectively evaluated the impact of interruption during radiotherapy on survival in patients with NPC and found that more interruption during radiotherapy led to worse outcomes [17]. Jolfaie et al. conducted a systemic review focusing on the effect of glutamine intake on complications of colorectal and colon cancer treatment and showed that glutamine supplementation improved complications induced by cancer therapeutic methods and shortened the length of hospital stay [18]. Second, the dipeptiven supplement provided nutritional support in NPC patients treated with CCRT. Dipeptiven is a parenteral amino acid solution containing dipeptide alanyl-glutamine, which is a precursor of protein as well as an energy source. Meng et al. investigated the impact of nutritional support among NPC patients receiving CCRT and demonstrated that early nutritional intervention brought survival benefits to NPC patients by maintaining well-nourished status and improving CCRT treatment compliance [19]. Another famous study published in 2010 also confirmed this conclusion. Patients with early palliative care, including nutrition support, received less aggressive care at the terminal stage but attained longer survival. [20] Taken together, dipeptiven supplementation is crucial in NPC patients treated with CCRT. Further prospective clinical trials are warranted to validate our results.

The Multinational Association of Supportive Care in Cancer and International Society of Oral Oncology (MASCC/ISOO) conducted systematic reviews regarding various management strategies for OM and established clinical practice guidelines based on current evidence [21]. Current guidelines suggest that head and neck cancer patients receiving CCRT need glutamine for OM prophylaxis. This recommendation was drawn according to two randomized controlled trials [22,23]. Taken together, glutamine supplementation significantly diminished the severity of OM and cancer pain, as well as reducing their duration. Several preceding articles in the literature also confirmed the role of glutamine in the prevention and management of OM among cancer patients [24]. Furthermore, previous studies showed that glutamine supplementation can reduce AE from chemotherapy or radiotherapy and that it had a protective effect that was associated with improved survival [25]. Glutamine supplementation also can lower the incidence of opioid usage, tubal feeding, hospitalization due to adverse effects, and treatment interruption caused by OM [26]. Our study also had similar conclusions. Patients with glutamine supplementation had fewer grade 3–4 AEs. We believe that a glutamine supplement may ameliorate toxicity, improve quality of life, and augment treatment efficacy.

However, safety issues of glutamine supplementation in cancer cell proliferation had been raised for decades. A more recent study in the literature investigating glutamine metabolism in cancer cell lines found that cancer cells indeed demonstrated the highest glutamine uptake as well as glucose consumption [27]. This might limit the clinical utility of glutamine in cancer patients. Nevertheless, these publications were only cell line studies, not human studies. The application of these cell line studies to humans is uncertain. To date, there has been a lot of clear evidence that glutamine supplementation can improve treatment toxicity. However, there are no solid data that glutamine might compromise the survival benefits. Based on our retrospective study, a glutamine supplement enhanced treatment efficacy and prolonged the oncologic outcomes. Further randomized studies are needed to confirm our results. 

Our study was a retrospective observational study with several inevitable possibilities of selection bias. Dipeptiven supplementation was at the discretion of physicians, rather than under randomized control. This might be a major bias in this study. Moreover, a small number of patients, a single institutional experience, and an inconsistent follow-up interval were also limitations of our study. Our study demonstrated the positive impact of parenteral glutamine supplementation in NPC patients treated with CCRT. In spite of several limitations inherent to retrospective studies, our study paves the way toward the management of AE in NPC patients. Given that there are no prospective randomized controlled trials with larger sample sizes, our retrospective observational study could provide evidence for physicians who treat NPC patients receiving CCRT.

## 5. Conclusions

Our study investigated the impact of parenteral glutamine (dipeptiven) supplementation on oncologic outcomes in NPC patients treated with CCRT. Based on our results, we disclosed that a dipeptiven supplement was crucial in NPC patients treated with CCRT. The dipeptiven supplement resulted in a lower CCRT interruption rate and SAE rate. Differences in the ORR and DCR were insignificant in both arms, but the CR rate was significantly higher in the dipeptiven (+) arm. The median OS was not reached in the dipeptiven (+) group, and it was 30 months in the dipeptiven (–) group. In our multivariate analysis, dipeptiven supplementation and CCRT interruption were independent predictor associated with better survival. The OS was longest in patients with a dipeptiven supplement and patients who had CCRT interruption had significantly worse OS. As for safety profiles, grade 3–4 AEs were fewer in dipeptiven (+) than in dipeptiven (–). These conclusions are clinically valuable for the management of NPC patients treated with CCRT. Further prospective randomized controlled trials are warranted to validate our conclusions.

## Figures and Tables

**Table 1:** Baseline clinical characteristics of 144 patients with nasopharyngeal carcinoma, stratified by dipeptiven.

	Dipeptiven (+)		Dipeptiven (–)		*p*
	*N* = 41		*N* = 103		
Gender					0.73
Male	31	76%	75	73%	
Female	10	24%	28	27%	
Age					0.61
≦60 years	30	73%	71	69%	
>60 years	11	27%	32	31%	
ECOG PS					0.85
0–1	35	85%	90	87%	
2	6	15%	13	13%	
BMI					0.52
≦24 kg/m^2^	14	34%	41	40%	
>24 kg/m^2^	27	66%	62	60%	
Weight loss					0.60
≦5%	36	88%	87	84%	
>5 %	5	12%	16	16%	
Renal function					0.91
CCr > 60 mL/min	39	95%	99	96%	
CCr ≦ 60 mL/min	2	5%	4	4%	
T stage					0.44
1–2	22	54%	48	47%	
3–4	19	46%	55	53%	
N stage					0.71
0–1	13	32%	36	35%	
2–3	28	68%	67	65%	
Clinical stage					0.77
I–II	8	20%	18	17%	
III–IVA	33	80%	85	83%	
Induction chemotherapy					0.98
No	29	71%	73	71%	
Yes	12	29%	30	29%	
Adjuvant chemotherapy					0.62
No	11	27%	24	23%	
Yes	30	74%	79	77%	

ECOG PS, Eastern Cooperative Oncology Group Performance Status; BMI, body mass index; CCr, creatinine clearance rate.

**Table 2:** Oncologic outcomes of 144 patients with nasopharyngeal cancer receiving concurrent chemoradiotherapy, stratified by dipeptiven.

	Dipeptiven (+) *N* = 41	Dipeptiven (–) *N* = 103	*p*
CCRT interruption rate	0%	22 (21%)	<0.01
SAE rate	0%	12 (12%)	0.02
CR (%)	32 (78%)	59 (57%)	0.02
PR (%)	9 (22%)	34 (33%)	
SD (%)	0 (0%)	6 (6%)	
PD (%)	0 (0%)	4 (4%)	
ORR (%)	41 (100%)	93 (90%)	0.03
DCR (%)	41 (100%)	99 (96%)	0.20
mOS (m)	NR	30	<0.01

CCRT, concurrent chemoradiotherapy; SAE, severe adverse effect; CR, complete response; PR, partial response; SD, stable disease; PD, progressive disease; ORR, objective response rate; DCR, disease control rate; mOS, median overall survival.

**Table 3:** Cox regression analysis of parameters associated with overall survival.

	Univariate		Multivariate	
Variables	HR (95% CI)	*p* Value	HR (95% CI)	*p* Value
Gender, female vs. male	0.52 (0.22–1.23)	0.13		
Age, ≦60 vs. >60	0.74 (0.39–1.38)	0.33		
ECOG PS, 0–1 vs. 2	0.78 (0.41–1.47)	0.41		
BMI, ≦24 vs. >24	0.48 (0.26–0.89)	0.02	0.68 (0.36–1.27)	0.22
Weight loss, ≦5% vs. >5%	0.80 (0.44–1.12)	0.34		
CCr, >60 vs. ≦60	0.82 (0.32–1.55)	0.25		
T stage, 1–2 vs. 3–4	0.91 (0.49–1.69)	0.76		
N stage, 0–1 vs. 2–3	0.79 (0.42–1.49)	0.45		
Clinical stage, I–II vs. III–IVA	0.45 (0.16–1.26)	0.12		
Induction chemotherapy, yes vs. no	0.10 (0.01–0.74)	0.02	0.13 (0.02–0.95)	0.04
Adjuvant chemotherapy, yes vs. no	0.21 (0.11–0.40)	<0.01	0.65 (0.32–1.32)	0.23
Dipeptiven, yes vs. no	0.13 (0.04–0.44)	<0.01	0.31 (0.09–0.95)	0.04
CCRT interruption, no vs. yes	0.15 (0.08–0.29)	<0.01	0.32 (0.15–0.68)	<0.01

HR, hazard ratio; CI, confidence interval; ECOG PS, Eastern Cooperative Oncology Group Performance Status; CCRT, concurrent chemoradiotherapy.

**Table 4:** Grade 3 to 4 treatment-related adverse effects in 144 nasopharyngeal cancer receiving concurrent chemoradiotherapy, stratified by dipeptiven.

	Dipeptiven (+) *N* = 41	Dipeptiven (–) *N* = 103	*p*
Hematologic events, *n* (%)			
Neutropenia	1 (2%)	16 (16%)	0.02
Febrile neutropenia	0 (0%)	4 (4%)	0.20
Anemia	1 (2%)	8 (8%)	0.23
Non-hematologic events, *n* (%)			
Fatigue	3 (7%)	25 (24%)	0.02
Anorexia	2 (5%)	15 (15%)	0.08
Diarrhea	0 (0%)	1 (1%)	0.52
Vomiting	3 (7%)	8 (8%)	0.92
Oral mucositis	2 (5%)	21 (20%)	0.02
Hearing impairment	0 (0%)	2 (11%)	0.36
Peripheral neuropathy	1 (4%)	12 (22%)	0.03

## Data Availability

All data are available upon request to the corresponding author.
